# Let's Talk About Sex: Improving Measurement of Contraceptive Use in Cross-Sectional Surveys by Accounting for Sexual Activity Recency

**DOI:** 10.9745/GHSP-D-21-00597

**Published:** 2022-04-28

**Authors:** Suzanne O. Bell, Elizabeth Larson, Shannon N. Wood, Performance Monitoring for Action Principal Investigators Group

**Affiliations:** a Department of Population, Family and Reproductive Health, Johns Hopkins Bloomberg School of Public Health, Baltimore, MD, USA.; b Contributing members listed at the end of the article.

## Abstract

**Introduction:**

Standard measures of contraceptive prevalence have known biases given that they do not reflect sexual activity and true exposure to the risk of an unintended pregnancy. In this study, we aim to understand the extent to which women protect themselves against unintended pregnancy, taking into account exposure to sex, by examining contraceptive use patterns by marital status and recent sexual activity.

**Methods:**

Data come from population-based surveys of reproductive age women in 9 low-resource settings. We estimated contraceptive prevalence using (1) the standard current contraceptive use measure, (2) a new measure of contraceptive use at last sex, and (3) a comprehensive measure that combines current use and use at last sex. Analyses are stratified by site and examine patterns by marital status only, and by both marital status and sexual activity separately. We then examined method mix by each contraceptive measure.

**Results:**

Study findings reveal distinct patterns in contraceptive use in relation to marital status and sexual recency across sites. Overall, married women tended to report higher levels of current contraceptive use compared to use at last sex, whereas unmarried women reported higher levels of contraceptive use at last sex. When examining these measures by sexual activity and marital status, results indicate lower levels of contraceptive use among women who had not had sex in the month prior to the survey, for both married and unmarried women. The comprehensive measure of contraceptive use yielded the highest estimates, by design. Method mix varied consistently by contraceptive measure, with current use tending to capture more permanent and long-acting methods and use at last sex more likely to capture short-acting and coital-dependent methods.

**Discussion:**

These findings have important implications for how the family planning field evaluates unintended pregnancy risk and unmet need for contraception within low-resource settings, given different estimates yield discrepant estimates for who is “at risk.”

## Abstract (precis)

Findings suggest that the contraceptive use of unmarried women and those who were not recently sexually active are less likely to be captured in standard measures of current contraceptive use. Incorporating information from questions about contraceptive use at last sex may better capture coital-dependent method use and provide a more accurate assessment of who is protected against an unintended pregnancy at next sex.

## Teaser

**Key Findings:**

- Married women tended to report higher levels of current contraceptive use compared to use at last sex, whereas unmarried women reported higher levels of contraceptive use at last sex.
- When examining these measures by sexual activity and marital status, results indicate lower levels of contraceptive use among women who had not had sex in the month before the survey, for both married and unmarried women.
- The contraceptive method mix produced by these measures confirm the presence of systematic biases in standard measures of contraceptive use, with current users more likely to report longer-acting methods compared to women who reported using at last sex, who were more likely to report coital-dependent modern methods.

**Key Implications:**

- Study findings highlight the need for family planning providers to consider coital frequency when counseling women on contraceptive methods. Thorough contraceptive counseling can ensure women are aware of their options and can best exercise their preferences whenever they next have sex.
- Our new comprehensive contraceptive use measure may provide a more accurate assessment of a woman's protection against unintended pregnancy at next sexual encounter, which is useful for national and global monitoring efforts, especially as the share of women who are sexually active and unmarried increases around the world.

## INTRODUCTION

Understanding the family planning (FP) needs of all women is essential for ensuring they have reproductive autonomy and can effectively achieve their reproductive goals. FP programs and policies have long measured contraceptive prevalence and unmet need for contraception as a means of monitoring women's needs, and these measures are used to assess progress toward global goals, like FP2020 (now FP2030) and the 2030 Sustainable Development Goals.^1^ These global FP efforts are motivated by the link between contraceptive use, risk of unintended pregnancy, and associated negative sequelae.^2^^–^^5^ Historically, these indicators were measured only among married women under the assumption that the majority of sexual activity, and therefore contraceptive use, occurred within marital partnerships. However, as the global youth population grows larger, age at first marriage increases, and the age at sexual debut remains relatively constant, sexual activity is increasingly occurring outside of marriage.^6^^–^^8^ These changes have been reflected in the move toward measuring contraceptive prevalence among all reproductive-aged women, regardless of marital status.

A related assumption, particularly regarding unmet need, is that sexual activity among married couples occurs regularly. Yet qualitative data asserts that in contexts where sex is viewed primarily for procreation, frequency is likely to decrease as ideal family sizes are met.^9^ Additionally, male economic migration and associated spousal separation may be reducing the frequency of marital sexual activity in many settings.^10^ Therefore, the assumptions about who is in need of contraception may be increasingly inaccurate. Traditional measures of contraceptive prevalence and unmet need may not be well-positioned to capture the extent to which women's contraceptive needs are being met or the extent to which women are protected against unintended pregnancy.

Prior research has revealed significant differences in contraceptive prevalence depending on how one defines the population at risk. In particular, restricting the population to fecund, nonpregnant women and by sexual activity recency results in large increases in contraceptive prevalence, particularly among unmarried women across low-, middle-, and high-resource settings.^11^^,^^12^ In the United States, contraceptive prevalence was 62% among all women but 86% among fecund, nonpregnant women who had sex in the last 4 weeks; the results were similar in low-income countries, where the difference in estimates was 23 percentage points.^11^ In both settings, the difference was greatest among unmarried women. Similar patterns emerged from a more recent study examining the impact of using different sexual recency periods, finding that contraceptive prevalence is systematically lower and unmet need systematically higher among unmarried women as time since last sex increases; estimates are relatively similar among married women.^12^ Authors ultimately recommended measuring contraceptive prevalence and unmet need among those who had sex in the last month to produce the most precise measure of contraceptive needs.^12^ Studies of unmet need also reveal the importance of sexual activity, finding that women who are not using contraception are less likely to have had sex recently.^13^^,^^14^

Other research has raised issues with the standard contraceptive use question.^11^ Results from the United States suggest distinct benefits in asking specifically about contraceptive use at last sex as opposed to just current contraceptive use.^11^ Findings reveal biases in the types of methods captured on these different questions, with short-acting methods tending to be omitted in response to questions on current use, while long-acting and permanent methods are less likely to be mentioned when referring to use at last sex.^11^ Resulting estimates of contraceptive use were similar for currently married women, as well as for those who had sex recently (regardless of marital status), but the difference between the use at last sex and the current contraceptive use estimates increased as the time since last sex increased, particularly for those not married.^11^ No equivalent data have explored this issue of contraceptive use at last sex and biases in method mix in low-resource settings, where greater dependence on traditional and coital dependent methods may exacerbate reporting biases observed in the United States. Existing research has already found that the current contraceptive use question often misses these methods in low-resource settings.^15^^–^^18^ Authors from prior studies suggest investigators address this issue in FP surveys by asking about contraceptive use at last sex and the specific method used to better understand contraceptive prevalence and use among women who are actually at risk of pregnancy.^12^

No equivalent data have explored this issue of contraceptive use at last sex and biases in method mix in low-resource settings.

To address this knowledge gap and better understand the extent to which women are able to protect themselves against unintended pregnancy—taking into account exposure to sex—we leveraged an existing data collection platform, using data from Burkina Faso, Côte d'Ivoire, the Democratic Republic of the Congo (DRC), Kenya, Niger, Nigeria, and Uganda. The first aim of this study is to assess contraceptive use relying on 3 different measures: (1) the traditional measure of current contraceptive use; (2) contraceptive use at last sex; and (3) a composite measure of both, which we are calling comprehensive contraceptive use. The second aim is to explore how the dynamics between these 3 measures vary by marital status and recent sexual activity. The third aim is to evaluate the distribution of contraceptive method use according to the current contraceptive use and use at last sex measures and identify the method mix among those who report use at last sex but not current use.

## METHODS

We used data from the Performance Monitoring for Action (PMA) project, which recently enrolled reproductive-age women in 7 countries in sub-Saharan Africa into a panel to monitor contraceptive and sexual and reproductive health indicators and dynamics over time.^19^ Nationally representative surveys in Burkina Faso, Côte d'Ivoire, Kenya, Niger, and Uganda and subnationally representative surveys in Kano and Lagos, Nigeria, and Kinshasa and Kongo Central, Democratic Republic of Congo (DRC) were conducted between November 2019 and March 2021; analyses used the baseline survey from each country panel. The PMA survey methodology employs a multistage cluster sampling design with probability proportional to size sampling of enumeration areas, randomly selecting 35 households from each enumeration area.^20^ Interviewers invited all women aged 15 to 49 years in each household to participate in the female survey.

The survey included 2 sets of questions on contraceptive use and methods in each setting, which were used to create 3 binary variables for contraceptive use. The first item used the standard Demographic and Health Survey (DHS) and PMA language: “Are you or your partner currently doing something or using any method to delay or avoid getting pregnant?” If the respondent replied “yes,” she was asked a follow-up question about the method(s) she is currently using. The second item used the language suggested by Fabic and Jadhav^12^: “The last time you had sex, did you or your partner use any method to avoid or prevent a pregnancy?,” followed by a question about the specific method(s), if affirmative. To more fully assess a woman's ability to prevent unintended pregnancy when she has sex, we created a third composite measure that combined reported current contraceptive use and contraceptive use at last sex, such that an affirmative response to either question would be categorized as “yes” for the comprehensive measure for a given woman; we refer to this measure as the comprehensive contraceptive use measure. We view this comprehensive measure as perhaps more indicative of a woman's likely protection against an unintended pregnancy the next time she has sex, accounting for the aforementioned biases associated with the individual contraceptive use measures.

We view this comprehensive measure as more indicative of a woman's likely protection against an unintended pregnancy the next time she has sex, accounting for the aforementioned biases associated with the individual contraceptive use measures.

Our analytic sample was restricted to nonpregnant, presumably fecund (not menopausal, no hysterectomy) women who had ever had sex (i.e., women who were potentially at risk of pregnancy and could need contraception). We did not incorporate fertility intentions in this analysis of contraceptive prevalence since this information would make the measure more akin to measures of met and unmet need. We first estimated the level of contraceptive use by each of the 3 contraceptive measures separately for each site. To explore the role of marital status (currently married/cohabiting—simply referred to as married—versus not) and recent sexual activity (sex in the last month or not), we estimated the prevalence of women who were married and who had sex recently and then stratified subsequent analyses of contraceptive use by (1) marital status only, and (2) both marital status and sexual activity. We calculated Wilson confidence intervals, which are more appropriate for proportions that lie between 0 and 1, particularly small proportions near zero where the normal distribution assumption of standard confidence intervals is not applicable given the proportion cannot be less than zero. Lastly, we examined the method mix among current users, users at last sex, current users who reported no contraceptive use at last sex, and current non-users who reported contraceptive use at last sex. To examine method mix, we combined methods into the following 5 categories: (1) sterilization (female and male); (2) long-acting reversible contraceptive (LARC—intrauterine device (IUD) and implant); (3) short-acting hormonal (pill, injectable, and N-tablet (Kenya only)); (4) coital-dependent modern (condoms, emergency contraception, diaphragm, and foam); and (5) “other” (cycle beads, lactational amenorrhea method, rhythm, withdrawal, and other). The “other” category includes both modern and traditional methods, however, we combined these methods given their similar behavioral features and lack of a consumable product. We conducted all analyses in Stata version 15.1, weighting analyses to account for the complex sampling design and calculating standard errors using the Taylor linearization method to adjust for clustering.

### Ethics Approval

Each woman provided informed consent before beginning the interview. Ethical review boards in each country provided approval for the study protocol. Further details on PMA survey methodology are available at www.pmadata.org/data/survey-methodology.

## RESULTS

Table 1 presents overall estimates of contraceptive use for each of the 3 measures (current contraceptive use, use at last sex, and comprehensive contraceptive use) by site. Among all women, measures of current contraceptive use were generally higher than measures of use at last sex, with the greatest absolute difference observed in Kongo Central (8.7 percentage points higher), followed by Côte d'Ivoire (6.2 percentage points). Conversely, Uganda (2.6 percentage points) and Kinshasa (0.9 percentage points) had **higher** estimates of contraceptive use at last sex compared to current use. The comprehensive contraceptive use measure was greater than the standard contraceptive use measure by between 0.6 percentage points higher in Kano and 9.0 percentage points in Uganda.

**TABLE 1.** Contraceptive Prevalence Using Current Use, Use at Last Sex, and a Composite Measure Among All Presumably Fecund Women Aged 15–49 Years Who Have Ever Had Sex, by Site

	Burkina Faso (N=5,016) % (95% CI)	Côte d'Ivoire (N=3,320) % (95% CI)	DRC: Kinshasa (N=1,950) % (95% CI)	DRC: Kongo Central (N=1,555) % (95% CI)	Kenya (N=7,380) % (95% CI)	Nigeria: Kano (N=738) % (95% CI)	Nigeria: Lagos (N=1,126) % (95% CI)	Niger (N=851) % (95% CI)	Uganda (N=2,922) % (95% CI)
Current contraceptive use	35.4 (31.8, 39.1)	36.34 (32.9, 39.7)	57.24 (53.2, 61.1)	45.74 (38.7, 52.9)	58.84 (56.9, 60.6)	14.04 (9.6, 19.9)	48.84 (44.9, 52.6)	31.74 (27.7, 35.9)	46.94 (42.6, 51.2)
Contraceptive use at last sex	30.44 (26.6, 34.6)	30.14 (26.5, 34.0)	58.14 (54.3, 61.8)	37.04 (30.8, 43.7)	55.04 (52.3, 57.6)	10.04 (6.1, 16.0)	44.54 (40.6, 48.4)	25.14 (20.4, 30.5)	49.54 (45.3, 53.7)
Comprehensive contraceptive use	39.64 (35.8, 43.6)	39.84 (36.3, 43.3)	63.84 (59.8, 67.6)	50.84 (43.9, 57.6)	65.64 (63.7, 67.4)	14.64 (10.1, 20.6)	53.84 (50.0, 57.6)	34.04 (30.3, 38.0)	55.84 (52.4, 59.2)

Abbreviations: CI, confidence interval; DRC, Democratic Republic of the Congo.

Percent of women who were married and who had sex in the last month varied widely across sites (Table 2). More than 9 in 10 women in Kano were married (94.0%), followed by 86.0% in Burkina Faso, whereas only 49.5% of women were married in Kinshasa. Among all women, approximately 60.0% to 70.0% had sex in the month before the survey in most sites, while 84.3% of women in Kano had had sex recently. When exploring recent sexual activity by relationship status, we see greater percentage of recent sex among married women (approximately 80.0% to 90.0% of women, except for Burkina Faso at 63.2%) compared to those unmarried (approximately 30.0% to 40.0% of women, outliers being Kano and Niger at 20.9% and 8.8%, respectively).

**TABLE 2.** Marital Status and Sexual Recency of Presumably Fecund Women Aged 15–49 Years Who Have Ever Had Sex, by Site

	Burkina Faso (N=5,016) % (95% CI)	Côte d'Ivoire (N=3,320) % (95% CI)	DRC: Kinshasa (N=1,950) % (95% CI)	DRC: Kongo Central (N=1,555) % (95% CI)	Kenya (N=7,380) % (95% CI)	Nigeria: Kano (N=738) % (95% CI)	Nigeria: Lagos (N=1,126) % (95% CI)	Niger (N=851) % (95% CI)	Uganda (N=2,922) % (95% CI)
Currently married/cohabiting	86.04 (83.8, 88.0)	67.84 (64.6, 70.9)	49.54 (46.1, 52.9)	67.54 (62.4, 72.3)	68.54 (67.0, 70.1)	94.04 (90.7, 96.1)	71.74 (68.6, 74.7)	84.64 (80.4, 87.9)	67.04 (61.0, 72.5)
Had sex in last month									
All women	60.24 (56.3, 63.9)	66.44 (63.7, 68.9)	68.14 (64.9, 71.1)	72.24 (68.7, 75.4)	69.74 (68.2, 71.2)	84.34 (80.9, 87.2)	68.34 (65.0, 71.3)	68.44 (63.0, 73.3)	64.94 (58.6, 70.8)
Married/cohabiting	63.24 (58.9, 67.4)	76.44 (72.8, 79.6)	85.64 (83.4, 87.5)	85.04 (81.0, 88.3)	85.44 (83.9, 86.7)	88.44 (84.6, 91.4)	81.24 (78.2, 83.9)	79.34 (73.2, 84.3)	82.54 (79.0, 85.5)
Not married/cohabiting	41.54 (35.9, 47.2)	45.24 (41.7, 48.8)	50.94 (46.0, 55.9)	45.54 (39.4, 51.6)	35.54 (32.7, 38.4)	20.94 (7.3, 47.0)	35.54 (30.3, 41.1)	8.84 (4.2, 17.6)	29.24 (20.2, 40.3)

Abbreviations: CI, confidence interval; DRC, Democratic Republic of the Congo.

**FIGURE 1:**
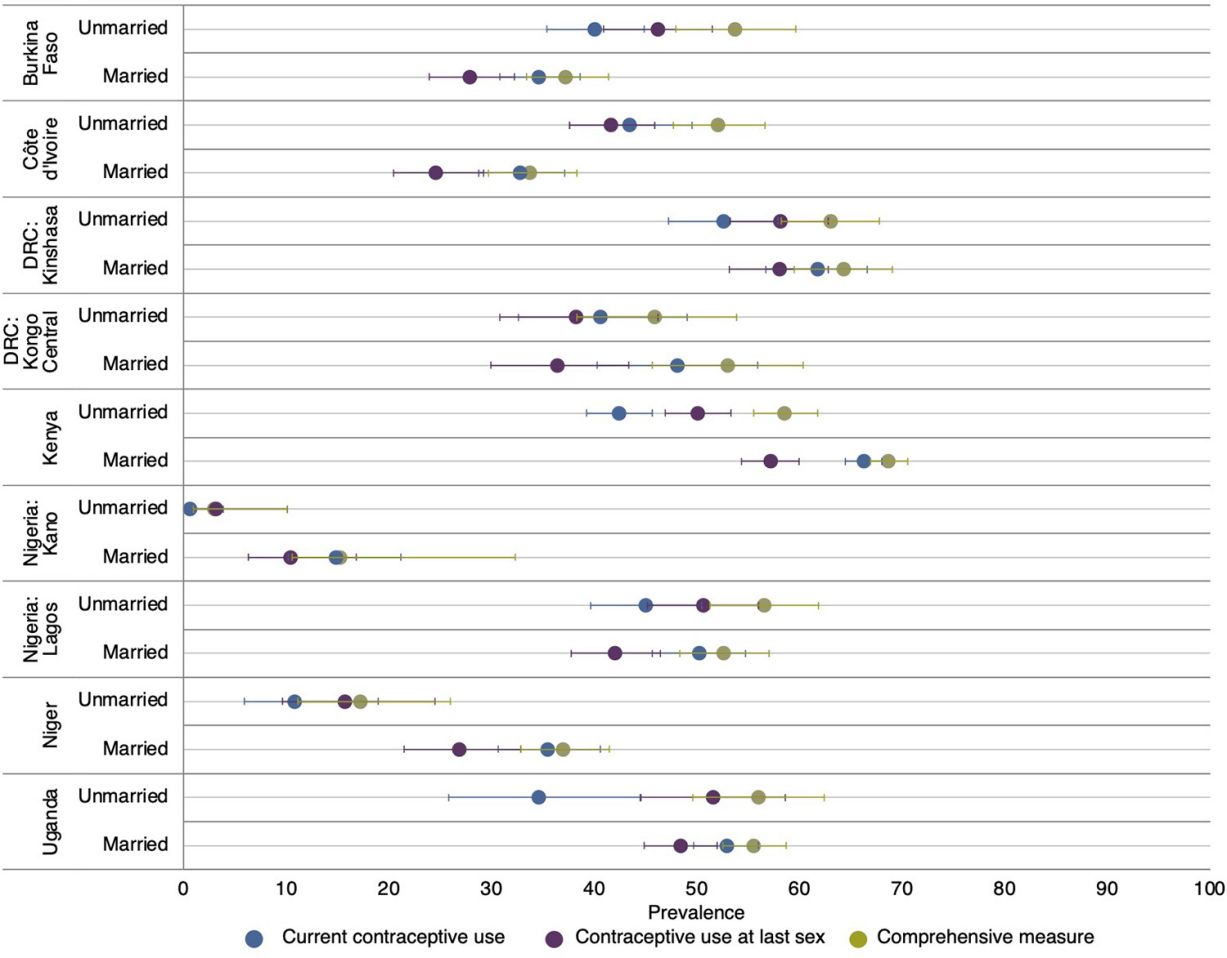
Contraceptive Prevalence Using Current Use, Use at Last Sex, and a Comprehensive Measure by Marital Status and Site Abbreviation: DRC, Democratic Republic of the Congo.

In Figure 1, we present the 3 contraceptive use measures stratified by marital status (Supplement Table 1 includes point estimates and sample sizes). These estimates reveal that the pattern of higher current contraceptive use compared to use at last sex was driven by married women, among whom the largest difference was approximately 9 percentage points in Kongo Central and Kenya. Unmarried women had the opposite pattern in nearly every site, with the measure of contraceptive use at last sex being higher than current use; this gap was 8 percentage points or greater in Burkina Faso, Kenya, Lagos, Niger, and Uganda. In each site, among both married and unmarried women, the comprehensive measure produced the highest estimate of contraceptive use, higher than the standard current contraceptive use measure by between 0.5 (Kano) and 5.0 (Kongo Central) percentage points among married women and 2.5 (Kano) and 21.6 (Uganda) percentage points among unmarried women.

These estimates reveal that the pattern of higher current contraceptive use compared to use at last sex was driven by married women.

**FIGURE 2:**
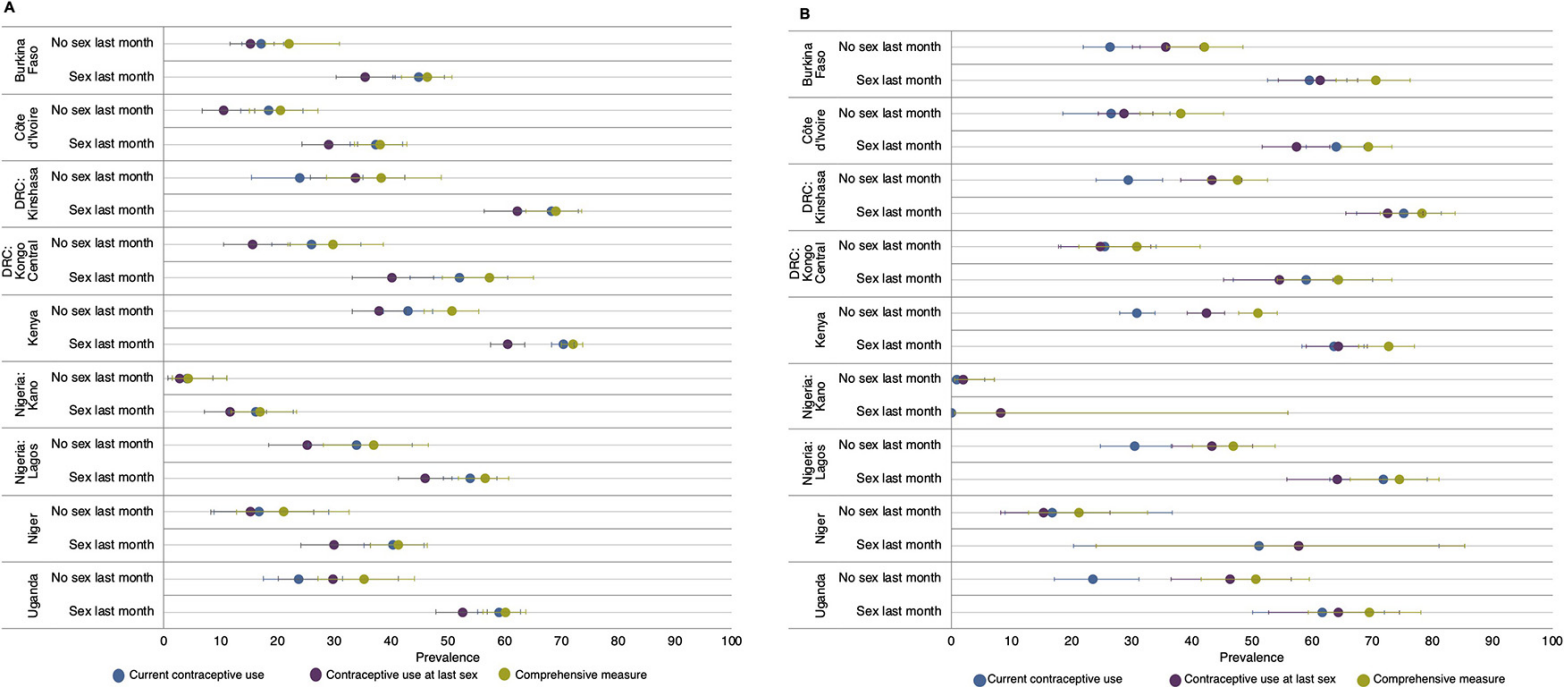
(A) Contraceptive Prevalence Using Current Use, Use at Last Sex, and a Comprehensive Measure by Whether Woman Had Sex in Last Month, and Site, Among Married Women; (B) Contraceptive Prevalence Using Current Use, Use at Last Sex, and a Comprehensive Measure by Whether Woman Had Sex in Last Month, and Site, Among Unmarried Women Abbreviation: DRC, Democratic Republic of the Congo.

In Figure 2, we disaggregate the estimates by whether the woman reported having had sex in the last month and whether she was currently married (Figure 2a) or not (Figure 2b) (Supplement Table 2 includes point estimates and sample sizes). In general, we observed substantially higher levels of contraceptive use across sites for all measures among women who were sexually active within the past month. In most sites, contraceptive prevalence was approximately 2 times greater for women who had sex in the last month compared to those who did not have sex recently, regardless of marital status, ranging from 1.4 to 4.4 times greater; in no site was current contraceptive use lower for women who recently had sex compared to those who had not. The pattern was similar for the use at last sex as well as comprehensive measures, although the prevalence among those who were recently sexually active compared to those who were not was slightly closer for the comprehensive measure. Among married women in both sexual activity groups, we found higher current contraceptive use levels compared to contraceptive use at last sex (except for not sexually active women in Kinshasa and Uganda). We observed somewhat larger differences in these measures for women who had had sex in the last month (ranging from 5 percentage points in Kano to 9 percentage points in Burkina, Kenya, and Kongo Central) compared to those who had not had sex recently (ranging from 2 percentage points in Burkina and Kano to 10 percentage points in Kongo Central). In comparison, among unmarried women, we saw much more heterogeneity in these measures and associated differences, particularly among those who were recently sexually active. In contrast to married women, unmarried women had larger differences in the contraceptive use measures between current use and use at last sex among those who had **not** had sex in the last month (ranging from 1 percentage point lower in Kongo Central and Kano to more than 10 percentage points lower in Burkina Faso, Kinshasa, Kenya, Lagos, and Uganda) compared to unmarried women who had sex in the last month (ranging from 8 percentage points higher in Kano to 7 in Lagos).

**FIGURE 3:**
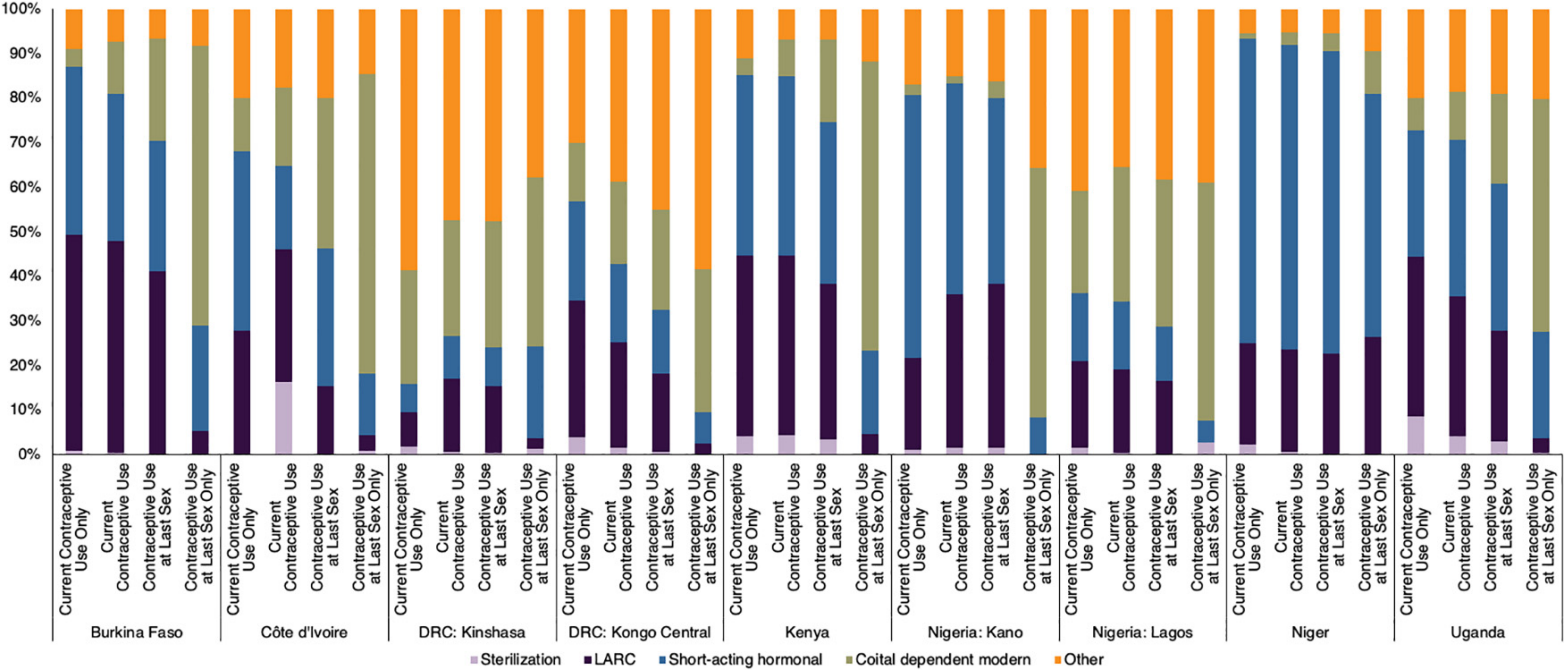
Contraceptive Method Mix Among Women Using Contraception According to Current Use and At Last Sex Measures, by Site Abbreviations: DRC, Democratic Republic of the Congo; LARC, long-acting reversible contraceptive.

Across sites, the method mix varied consistently between women who reported currently using contraception, using contraception at last sex, and those who reported using contraception at last sex but not currently (Figure 3) (Supplement Table 3 includes exact estimates and sample sizes). In general, we found that current users were more likely to report longer-acting methods like sterilization, LARCs, and to a lesser extent, short-acting hormonal contraceptives, compared to women who reported using at last sex. The exception was Kinshasa, where approximately 60% of current users reported “other” methods. Women who reported current contraceptive use but **not** use at last sex were more likely to report sterilization or LARCs and/or less likely to report coital dependent methods in many sites, including Burkina Faso, Kongo Central, Lagos, Niger, and Uganda. Those who reported use at last sex reported higher proportions of coital-dependent modern methods compared to those who reported current contraceptive use. In Kongo Central and Niger, and to a lesser extent Lagos and Kano, we also saw an increase in “other” methods among users at last sex. Among women who reported use at last sex but did not report current contraceptive use at the time of the survey, the aforementioned differences in method mix were even greater, with this group overwhelmingly using coital-dependent modern methods (greater than 50% in each site except for Kinshasa, Kongo Central, and Niger).

## DISCUSSION

Study findings reveal distinct patterns in contraceptive use in relation to marital status and sexual activity recency across study sites. Overall, married women tended to report higher levels of current contraceptive use compared to use at last sex, whereas unmarried women reported higher levels of contraceptive use at last sex. When examining these measures by sexual activity and marital status, results indicate lower levels of contraceptive use among women who had not had sex in the month prior to the survey, for both married and unmarried women. Consistent with prior literature,^11^ when examining the current use measure, women were more likely to report using longer-acting methods, whereas when asked about use at last sex, women focused more on coital-dependent methods, yielding substantial differences in method mix estimates. This discrepancy may partially be explained by the reason for contraceptive use, with the use at last sex question better capturing contraceptive use motivated by HIV or sexually transmitted infection protection, which is still relevant for understanding pregnancy risk. These findings have important implications for how the FP field evaluates unintended pregnancy risk and unmet need for contraception within lower-resource settings, given different estimates yield discrepant estimates for who is “at risk.” Additionally, a thorough understanding of the types of methods women are using is crucial to ensure national and international policy makers' contraceptive provisions are consistent with women's preferred use.

Our findings have important implications for how the FP field evaluates unintended pregnancy risk and unmet need for contraception within lower-resource settings, given different estimates yield discrepant estimates for who is “at risk.”

The difference in contraceptive use was more prominent when stratifying by sexual recency than by marital status, which is in line with previous findings highlighting the importance of sexual recency when measuring contraceptive prevalence.^11^^,^^12^ Our results further align with prior research from high-resource settings, suggesting the biases in contraceptive method reporting by contraceptive use question (current versus last sex) are also present in lower-resource settings.^11^ Given findings indicate particularly different levels of protection against pregnancy for unmarried women by contraceptive measurement approach, our results emphasize the value of asking about contraceptive use at last sex. These findings have implications for our understanding of protection against pregnancy among adolescents as they are more likely to be unmarried and having infrequent sex, thus not always necessitating continuous contraceptive use. Standard measures of contraceptive prevalence, which look at current use, will therefore suggest adolescents are at a greater risk of pregnancy than actually exists for them. Future longitudinal research should examine how use at last sex predicts future contraceptive use and pregnancy risk among this population. We recommend that large, nationally, or regionally representative surveys incorporate this item, which DHS (in addition to PMA) recently adopted.

Our findings also have substantial implications for FP providers and highlight the need for consideration of coital frequency when counseling women on contraceptive methods. Thorough contraceptive counseling—inclusive of discussions on sexual health, frequency of sexual activity, and nature of relationships—can ensure women are aware of their options and can best exercise their preferences whenever they next have sex. As the nature of a woman's sexual relationships changes over her life course, contraceptive counseling must similarly adapt to ensure she is able to use the most appropriate method for her and her relationship.

### Limitations

While this study makes important contributions to our understanding of contraceptive use measurement, it is not without limitations. Small sample sizes among unmarried women, particularly in Kano and Niger, and married women who did not have sex in the last month (Kinshasa, Kongo Central, Kano, and Lagos) limited our precision for subgroup analyses. Further, in some contexts, particularly rural areas where sexual activity and discussions of such are still taboo for unmarried adolescents, we may face underreporting of sexual activity and contraceptive use among unmarried respondents. Additionally, the use at last sex question asks only whether the respondent or her partner “used any method” whereas the current use measure asks about “doing something or using any method” to delay or avoid pregnancy. The narrower reference to “method” only without language related to “doing something” in the use at last sex question may result in continued underreporting of traditional and non-commodity modern method use (“other” methods in our analysis).

## CONCLUSION

Our new comprehensive contraceptive use measure may provide a more accurate assessment of a woman's protection against unintended pregnancy at next sexual encounter. We know both current contraceptive use and use at last sex measures are biased in different ways—a comprehensive measure may help to address both of these measures' biases; however, it is unclear to what extent. Further research is needed to continue to understand the accuracy of these measures across contexts and time and their relation to contraceptive needs, preferences, and unintended pregnancy. While outside the scope of this article, future work should also use the comprehensive contraceptive use measure to recalculate unmet need and compare estimates to the standard approach. The relative consistency of findings in the present results across different cultural contexts, with varying levels of contraceptive use and distinct method patterns, bolsters the robustness of our findings. We recommend investigators measuring contraceptive use consider the biases in contraceptive reporting, particularly among those who have not had sex recently, and ask about use at last sex to combine this information with that obtained via the standard current use question. By design, our comprehensive contraceptive use measure will always be equal to or greater than the standard contraceptive use measure since it incorporates additional information based on contraceptive use at last sex. Using our proposed measure would thus mean reevaluating existing FP and contraceptive use programs and policy goals in light of the likely higher levels of contraceptive use indicated by the new measure. Resulting estimates, while not necessarily a true prevalence of contraceptive use, may provide a more accurate assessment of women's protection against unintended pregnancy. This new comprehensive contraceptive use measure is useful for global monitoring efforts, especially as the share of women who are sexually active and unmarried grows and the extent of spousal separation as a result of economic migration increases, populations for which standard measures of contraceptive use may be inadequate at assessing pregnancy risk.

## Supplementary Material

GHSP-D-21-00597-supplement.pdf

## Notes

**Peer Reviewed**

**First published online:** April 19, 2022.
