# Supplementary material for: Let's Talk About Sex: Improving Measurement of Contraceptive Use in Cross-Sectional Surveys by Accounting for Sexual Activity Recency
Source: Glob Health Sci Pract. 2022 Apr 28;10(2):e2100597. doi: 10.9745/GHSP-D-21-00597 (PMC9053161; doi:10.9745/GHSP-D-21-00597)
Supplement: GHSP-D-21-00597-supplement.pdf [file GHSP-D-21-00597-supplement.pdf]

Supplement to: Bell S, Larson E, Wood SN; PMA Principal Investigators Group. Let's talk about sex: improving measurement of contraceptive use in cross-sectional surveys by accounting for sexual activity recency. *Glob Health Sci Pract*. 2021;10(2): e2100597. https://doi.org/10.9745/GHSP-D-21-00597

Supplement Table 1. Contraceptive Prevalence Using Current Use, Use at Last Sex, and a Comprehensive Measure by Marital Status, and Site

Appendix Table 1. Contraceptive prevalence using current use, use at last sex, and a comprehensive measure by marital status and site

	Burkina Faso			Cote d'Ivoire			DRC: Kinshasa			DRC: Kongo Central			Kenya			Nigeria: Kano			Nigeria: Lagos			Niger			Uganda		
	%	95% CI		%	95% CI		%	95% CI		%	95% CI		%	95% CI		%	95% CI		%	95% CI		%	95% CI		%	95% CI	
Married women	N=	3864		N=	2251		N=	997		N=	1005		N=	5033		N=	688		N=	795		N=	702		N=	1955	
Current contraceptive use	34.6	30.8	38.7	32.8	28.8	37.2	61.8	56.8	66.6	48.1	40.3	56.0	66.3	64.4	68.1	14.9	10.2	21.2	50.2	45.7	54.7	35.5	30.6	40.6	52.9	49.7	56.1
Contraceptive use at last sex	27.9	23.9	32.2	24.6	20.4	29.2	58.0	53.2	62.8	36.4	30.0	43.3	57.2	54.3	60.0	10.5	6.3	16.8	42.1	37.8	46.5	26.8	21.5	32.9	48.4	44.9	52.0
Comprehensive contraceptive use	37.3	33.4	41.4	33.9	29.7	38.3	64.4	59.5	69.1	53.1	45.6	60.4	68.8	67.0	70.6	15.3	10.6	21.7	52.7	48.3	57.1	37.1	32.9	41.5	55.7	52.6	58.7
Unmarried women	N=	1152		N=	1069		N=	953		N=	550		N=	2347		N=	50		N=	331		N=	149		N=	967	
Current contraceptive use	40.1	35.4	44.9	43.5	37.6	49.6	52.6	47.3	57.9	40.6	32.7	49.1	42.4	39.3	45.7	0.6	0.1	3.9	45.0	39.7	50.5	10.8	5.9	18.9	34.6	25.8	44.5
Contraceptive use at last sex	46.2	40.9	51.5	41.7	37.6	45.9	58.1	53.3	62.8	38.2	30.8	46.2	50.1	46.9	53.3	3.1	0.9	10.1	50.6	45.2	56.0	15.7	9.7	24.5	51.6	44.6	58.6
Comprehensive contraceptive use	53.9	48.0	59.7	52.2	47.7	56.6	63.2	58.3	67.8	46.0	38.4	53.9	58.7	55.5	61.8	3.1	0.9	10.1	56.6	51.3	61.9	17.3	11.1	26.0	56.1	49.6	62.4

Supplement to: Bell S, Larson E, Wood SN; PMA Principal Investigators Group. Let's talk about sex: improving measurement of contraceptive use in cross-sectional surveys by accounting for sexual activity recency. *Glob Health Sci Pract*. 2021;10(2): e2100597. https://doi.org/10.9745/GHSP-D-21-00597

Supplement Table 2. Contraceptive Prevalence Using Current Use, Use at Last Sex, and a Comprehensive Measure by Marital Status, Whether Woman Had Sex in Last Month, and Site

Appendix Table 2. Contraceptive prevalence using current use, use at last sex, and a comprehensive measure by marital status, whether had sex in last month, and site

	Burkina Faso			Cote d'Ivoire			DRC: Kinshasa			DRC: Kongo Centra			Kenya			Nigeria: Kano			Nigeria: Lagos			Niger			Uganda		
**Sex last month**	%	95% CI		%	95% CI		%	95% CI		%	95% CI		%	95% CI		%	95% CI		%	95% CI		%	95% CI		%	95% CI	
Married women	N= 2750			N= 1696			N= 846			N= 861			N= 4320			N= 606			N= 643			N= 556			N= 1614		
Current contraceptive use	44.8	40.3	49.4	37.3	32.8	42.0	68.2	63	73	52.0	43.4	60.5	70.3	68.3	72.2	16.3	11.4	22.7	54.0	49.3	58.6	40.4	35.2	45.8	59.1	55.3	62.8
Contraceptive use at last sex	35.3	30.3	40.6	28.9	24.2	34.1	62.2	56.4	67.6	40.1	33.1	47.6	60.5	57.4	63.5	11.5	7.1	18.0	46.0	41.3	50.7	29.9	24.1	36.4	52.4	47.9	56.9
Comprehensive contraceptive use	46.3	41.8	50.8	38.0	33.5	42.7	68.8	63.7	73.5	57.2	48.9	65.1	71.9	69.9	73.8	16.8	11.8	23.3	56.4	51.9	60.8	41.3	36.4	46.3	60.0	56.2	63.7
Unmarried women	N= 469			N= 490			N= 459			N= 253			N= 842			N= 9			N= 124			N= 13			N= 283		
Current contraceptive use	59.4	52.6	65.9	64.0	59.1	68.6	75.1	67.4	81.5	58.9	46.8	70	63.6	58.3	68.6	0.0	--	--	71.7	62.9	79.2	51.1	20.3	81.1	61.6	50.0	72.1
Contraceptive use at last sex	61.3	54.5	67.6	57.4	51.7	62.9	72.5	65.6	78.5	54.5	45.2	63.5	64.3	59.0	69.2	8.1	0.6	56.0	64.0	55.8	71.5	57.6	24.1	85.3	64.4	52.8	74.5
Comprehensive contraceptive use	70.5	64.0	76.4	69.3	64.9	73.3	78.2	71.3	83.8	64.3	54.2	73.3	72.6	67.7	77.1	8.1	0.6	56.0	74.4	66.3	81.0	57.6	24.1	85.3	69.5	59.4	78.0
**No sex last month**																											
Married women	N= 1114			N= 555			N= 151			N= 144			N= 713			N= 82			N= 152			N= 146			N= 341		
Current contraceptive use	17.1	13.7	21.1	18.4	13.5	24.4	23.9	15.5	35.1	26.1	19	34.6	42.9	38.7	47.3	4.2	1.5	11.0	34.0	25.5	43.7	16.7	8.9	29.0	23.7	17.4	31.5
Contraceptive use at last sex	15.2	11.7	19.5	10.5	6.73	16.1	33.6	25.9	42.4	15.5	10.5	22.2	37.8	33.1	42.6	2.6	0.8	8.6	25.2	18.5	33.4	15.2	8.3	26.4	29.7	20.2	41.2
Comprehensive contraceptive use	22.0	17.8	26.8	20.4	15	27.2	38.3	28.6	48.9	29.6	21.9	38.7	50.7	45.9	55.5	4.2	1.5	11.0	36.9	28.2	46.5	21.1	12.8	32.6	35.1	27.0	44.1
Unmarried women	N= 683			N= 579			N= 494			N= 297			N= 1505			N= 41			N= 207			N= 136			N= 684		
Current contraceptive use	26.4	22.0	31.3	26.6	18.6	36.5	29.3	24.1	35.1	25.4	18.3	34.1	30.8	27.9	33.9	0.8	0.1	4.8	30.4	24.8	36.6	6.9	3.3	14.1	23.4	14.7	35.1
Contraceptive use at last sex	35.5	30.1	41.3	28.7	24.4	33.5	43.2	38.2	48.3	24.7	17.8	33.1	42.3	39.3	45.4	1.8	0.5	7.1	43.3	36.7	50.1	11.6	6.3	20.5	46.4	36.5	56.5
Comprehensive contraceptive use	42.1	35.9	48.5	38.1	31.4	45.3	47.6	42.6	52.5	30.8	23.3	39.5	51.0	47.8	54.2	1.8	0.5	7.1	46.9	40.1	53.8	13.4	7.8	22.2	50.6	41.6	59.6

Supplement to: Bell S, Larson E, Wood SN; PMA Principal Investigators Group. Let's talk about sex: improving measurement of contraceptive use in cross-sectional surveys by accounting for sexual activity recency. *Glob Health Sci Pract*. 2021;10(2): e2100597. https://doi.org/10.9745/GHSP-D-21-00597

Supplement Table 3. Contraceptive Method Mix Among Women Using Contraception According to Current Use and Use At Last Sex Measures, by Site

Appendix Table 3. Contraceptive method mix among women using contraception according to current use and at last sex measures, by site

	Burkina Faso				Cote d'Ivoire				DRC: Kinshasa				DRC: Kongo Central			
	CCUO	CCU	CULS	CULSO	CCUO	CCU	CULS	CULSO	CCUO	CCU	CULS	CULSO	CCUO	CCU	CULS	CULSO
	%				%				%				%			
Sterilization	0.8	0.4	0.3	0.2	0.0	19.8	0.1	1.0	1.7	0.7	0.6	2.3	4.0	1.5	0.6	0.0
LARC	48.6	47.5	40.9	5.1	27.8	36.3	15.2	3.5	7.9	16.4	15.0	3.6	30.6	23.7	17.5	2.6
Short-acting hormonal	37.6	33.0	29.4	23.7	40.3	22.5	31.0	13.7	6.3	9.4	8.5	33.3	22.2	17.6	14.3	7.0
Coital dependent modern	4.1	11.9	22.8	62.8	11.9	21.4	33.8	67.2	25.5	26.1	28.3	60.8	13.2	18.5	22.6	32.1
Other	8.9	7.2	6.6	8.2	19.9	21.4	19.9	14.6	58.6	47.3	47.7	60.8	30.0	38.7	45.0	58.3
N	504	2155	1922	270	308	1116	949	134	99	1081	1134	133	187	767	660	75

	Kenya				Nigeria: Kano				Nigeria: Lagos				Niger				Uganda			
	CCUO	CCU	CULS	CULSO	CCUO	CCU	CULS	CULSO	CCUO	CCU	CULS	CULSO	CCUO	CCU	CULS	CULSO	CCUO	CCU	CULS	CULSO
	%				%				%				%				%			
Sterilization	4.2	4.5	3.6	0.3	1.1	1.5	1.6	0.0	1.6	0.4	0.2	2.9	2.4	0.7	0.0	0.0	8.6	4.2	2.9	0.3
LARC	40.5	40.1	34.9	4.5	20.7	34.4	36.8	0.0	19.5	18.8	16.4	0.0	22.7	23.0	22.8	26.4	35.9	31.3	25.0	3.4
Short-acting hormonal	40.4	40.3	36.3	18.6	59.1	47.4	41.5	8.3	15.1	15.2	12.2	4.8	68.4	68.3	67.7	54.4	28.1	35.3	33.0	23.9
Coital dependent modern	3.9	8.3	18.5	65.0	2.2	1.6	3.7	56.0	23.0	30.3	33.0	53.3	1.2	2.8	4.0	9.6	7.4	10.8	20.0	52.0
Other	11.0	6.8	6.7	11.7	17.0	15.1	16.3	35.7	40.8	35.3	38.3	39.0	5.4	5.2	5.5	9.5	19.9	18.5	19.1	20.3
N	791	4247	4003	514	34	125	96	5	101	545	509	58	74	274	221	21	182	1343	1431	257

CCUO = current contraceptive use only (not reported at last sex)
CCU = current contraceptive use
CULS = contraceptive use at last sex
CULSO = contraceptive use at last sex only (no current use reported)
